# Prenatal Diagnosis of 17p11.2 Copy Number Abnormalities Associated With Smith–Magenis and Potocki–Lupski Syndromes in Fetuses

**DOI:** 10.3389/fgene.2021.779237

**Published:** 2021-12-21

**Authors:** Meiying Cai, Xianguo Fu, Liangpu Xu, Na Lin, Hailong Huang

**Affiliations:** ^1^ Medical Genetic Diagnosis and Therapy Center, Fujian Maternity and Child Health Hospital, Affiliated Hospital of Fujian Medical University, Fujian Key Laboratory for Prenatal Diagnosis and Birth Defect, Fuzhou, China; ^2^ Department of Prenatal Diagnosis, Ningde Municipal Hospital, Ningde Normal University, Ningde, China

**Keywords:** smith-magenis syndrome, potocki-lupski syndrome, rare autosomal dominant, fetuses, copy number variant, SNP-array

## Abstract

Smith-Magenis syndrome and Potocki-Lupski syndrome are rare autosomal dominant diseases. Although clinical phenotypes of adults and children have been reported, fetal ultrasonic phenotypes are rarely reported. A retrospective analysis of 6,200 pregnant women who received invasive prenatal diagnosis at Fujian Provincial Maternal and Child Health Hospital between October 2016 and January 2021 was performed. Amniotic fluid or umbilical cord blood was extracted for karyotyping and single nucleotide polymorphism array analysis. Single nucleotide polymorphism array analysis revealed six fetuses with copy number variant changes in the 17p11.2 region. Among them, one had a copy number variant microdeletion in the 17p11.2 region, which was pathogenically analyzed and diagnosed as Smith-Magenis syndrome. Five fetuses had copy number variant microduplications in the 17p11.2 region, which were pathogenically analyzed and diagnosed as Potocki-Lupski syndrome. The prenatal ultrasound phenotypes of the six fetuses were varied. The parents of two fetuses with Potocki-Lupski syndrome refused verification. Smith-Magenis syndrome in one fetus and Potocki-Lupski in another were confirmed as *de novo*. Potocki-Lupski syndrome in two fetuses was confirmed to be from maternal inheritance. The prenatal ultrasound phenotypes of Smith-Magenis syndrome and Potocki-Lupski syndrome in fetuses vary; single nucleotide polymorphism array analysis is a powerful diagnostic tool for these diseases. The ultrasonic phenotypes of these cases may enrich the clinical database.

## 1 Introduction

The gene density of chromosome 17 is the second-highest in the human genome, and it contains a large number of chromosome deletion and duplication pathogenic regions (Zody et al., 2006; Goldenberg, 2018). It has several dose-sensitive genes, such as *PMP22* (Gillentine et al., 2018), *PAFAH1B1* (Chen et al., 2018), *YWHAE* (Mignon-Ravix et al., 2010), *RAI1* (Loviglio et al., 2016), and *NF1* (Margraf et al., 2019), which are involved in multiple genetic diseases (Shchelochkov et al., 2010). Smith-Magenis syndrome (SMS) and Potocki-Lupski syndrome (PTLS) result from microdeletion and microduplication, respectively, occurring at the same location in the 17p11.2 region, resulting in changes in the dose of sensitive genes and leading to two different syndromes (Heck et al., 2012; Twentyman, 2015).

SMS was first reported by Smith and colleagues in 1982 (Smith, 1982). It has an incidence of approximately 1:25,000 and is mostly sporadic. Currently, more than 100 cases have been reported worldwide, with affected individuals ranging in age from 1 month to 72 years (Edlman et al., 2007; Brendal et al., 2013). SMS is attributable to a 17p11.2 microdeletion. The main clinical manifestations in patients with SMS include mild to moderate intellectual impairment, cognitive impairment, distinct facial features (broad, square face, deep-set eyes, full cheeks, prominent jaw, flat nose, and downturned corners of the mouth), behavioral abnormalities, self-injurious behavior, and sleep disorders (Gouard et al., 2020). PTLS was first reported by Potocki and Lupski in 2000 (Potocki et al., 2007), and has an incidence of approximately 1:25,000 (Shuib et al., 2017). However, at present, less than 50 PTLS patients have been reported worldwide (Carter RD et al., 2013). PTLS is due to a 17p11.2 microduplication (Joseph et al., 2015). The main clinical manifestations in patients with PTLS include mild to moderate intellectual disability, short stature, inattention, autism, hyperactivity, triangular face, high zygomatic arch, frontal eminences, palatal dysplasia, and abnormal heart development (Twentyman, 2015).

SMS and PTLS are rare autosomal dominant diseases. Currently, clinical phenotypes of adults and children have been reported, while fetal ultrasonic phenotypes are rarely reported. In order to improve the understanding, diagnosis, and monitoring of these two rare genetic diseases in prenatal diagnosis, we retrospectively analyzed the ultrasonic phenotype and genetic results of one fetus with SMS and five fetuses with PTLS between October 2016 and January 2021 at the Fujian Provincial Maternal and Child Health Hospital.

## 2 Materials and Methods

### 2.1 Study Participants and Samples

A retrospective analysis of 6,200 pregnant women who received invasive prenatal diagnosis in the Fujian Provincial Maternal and Child Health Hospital from October 2016 to January 2021 was performed. The mean age of the pregnant women was 28.4 years (range, 17–46 years) and mean gestational age was 24.2 weeks (range, 16–38 weeks). Amniocentesis or umbilical cord blood puncture was performed according to the pregnant woman’s gestational age. Under the guidance of ultrasound, 25–30 ml of amniotic fluid was extracted from pregnant women with a gestational age of less than 28 weeks, of which 20 ml was used for chromosome karyotype analysis, and the remaining 5–10 ml was used for single nucleotide polymorphism array (SNP-array). For pregnant women over 28 weeks of gestation, 3 ml of umbilical cord blood was extracted under ultrasound guidance, of which 1.5 ml was used for chromosome karyotype analysis and 1.5 ml for SNP-array. All pregnant women received genetic counseling and signed informed consent forms prior to the invasive diagnostic procedures. This study was approved by the Medical Ethics Committee of the Fujian Provincial Maternal and Child Health Hospital (2014042) and was conducted in accordance with the Declaration of Helsinki.

### 2.2 Karyotype Analysis

Samples of amniotic fluid or umbilical cord blood were inoculated in 1,640 culture mediums (Hangzhou Bosheng Biotechnology, Hangzhou, China). The cord blood samples were cultured for 3 days, and the cells were harvested. The amniotic fluid samples were cultured for 8 days, and the cell morphology and fluid change were observed. Colchicine was added to the cells with good growth morphology in the growth peak period to make the cells undergo the metaphase of mitosis. Thereafter, the cells were harvested. Finally, Giemsa dye banding was conducted, the karyotype was collected by GSL-120 automatic chromosome scanning platform, and the karyotype was calculated and analyzed. The karyotypes were named according to the 2016 edition of the International System for Human Cytogenomic Nomenclature (ISCN), 40 karyotypes were counted for each case, and 5 were analyzed. To account for abnormalities, double karyotype counting and analysis were performed.

### 2.3 Single Nucleotide Polymorphism Array

DNA digestion, PCR, PCR purification, fragmentation, labeling, hybridization, washing, staining, and scanning were performed according to the standard operating procedure of Affymetrix CytoScan 750K GeneChip (Affymetrix, CA, United States). After scanning, CEL files were created to obtain the fluorescence intensity data. The Chromosome Analysis Suite (ChAS) v3.2 was used to perform a single sample analysis which compared the data in a CEL file to a previously created reference file to find genomic abnormalities. The SNP-array results were further analyzed in combination with relevant databases to determine the properties of the copy number variations (CNVs). The databases included the International Public DGV Benign Variation Database, Database of Genomic Variation (DGV), Database of Chromosomal Imbalance and Phenotype in Humans Using Ensembl Resources, Online Human Mendelian Genetic database, OMIM, the Cytogenomics Array Group CNV Database, NCBI, and PubMed. According to the guidelines of medical genetics in the United States (Riggs et al., 2020), CNVs are classified into pathogenic variants, possibly pathogenic variants, variants of uncertain clinical significance (VUS), possibly benign variants, and benign variants. In the fetuses with VUS, peripheral blood samples were collected from the parents for SNP-array, and family analysis was conducted to determine the exact classification of the CNV.

## 3 Results

### 3.1 Prenatal Ultrasound Characteristics of Fetuses

The ultrasound phenotype of fetus E3640 at 34 weeks of gestation was cardiac malformation, hyperhydramnios, and minor ultrasonographic markers. The ultrasound phenotype of fetus P3350 at 25 weeks of gestation was cardiac malformation. The ultrasound of P9874 at 24 weeks of gestation showed only minor ultrasonographic markers. The ultrasound phenotype of fetus R425 at 20 weeks of gestation was normal, but the mother displayed intellectual disability. The fetuses G8354 and P587 were from the same mother, and the mother displayed intellectual disability. G8354 was the first fetus in 2016 whose ultrasound phenotype showed cardiac malformation at 25 weeks of gestation. P587 was the second fetus in 2020 whose ultrasonic phenotype was normal at 22 weeks of gestation. The clinical information and prenatal ultrasound phenotypes of the six fetuses are shown in Table 1.

**TABLE 1:** Clinical information and prenatal ultrasound phenotypes of the six fetuses.

Case	Gestation	Specimen type	Mother phenotype	Father phenotype	Prenatal ultrasound characteristics
E3640	34	umbilical cord blood	normal	normal	VSD, minimal pulmonary valve regurgitation, persistent left superior vena cava, absence of nasal bone, thickened skin on the back of head and neck
G8354	25	amniotic fluid	intellectual disability	normal	VSD, severe tricuspid regurgitation, pericardial effusion, FGR
P587	22	amniotic fluid	intellectual disability	normal	normal
P9874	24	amniotic fluid	normal	normal	Strong echo points in left and right ventricular chordal tendineae, and a small amount of tricuspid regurgitation
P3350	25	amniotic fluid	normal	normal	Right aortic arch with mirrored branches, right ductus arteriosus
R425	20	amniotic fluid	intellectual disability	normal	normal

FGR, fetal growth restriction; VSD, ventricular septal defect. The fetuses G8354 and P587 were from the same mother, and the mother displayed intellectual disability.

### 3.2 Karyotype Analysis

Results of the karyotype analysis for all six fetuses were normal.

### 3.3 SNP-Array

**FIGURE 1:**
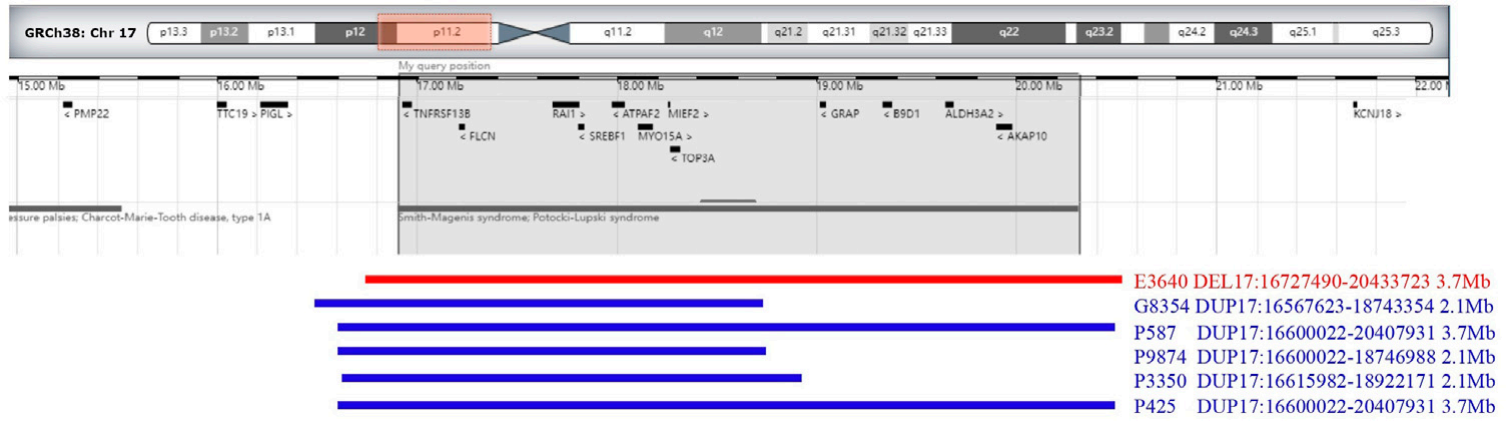
Chromosome 17p 11.2 imbalance detected by SNP-array. In fetus E3640, the SNP-array revealed that the microdeletion of the CNV in 17p11.2 involved a 3.7 Mb fragment and contained 38 OMIM genes (including *RAII*). In fetuses G8354, P587, P9874, P3350, and R425, the SNP-array revealed that he microduplication of the CNV in 17p11.2 involved about 2.1–3.7 Mb fragment size and contained 21–38 OMIM genes (including *RAII*).

The results of SNP-array analysis in the 6,200 fetuses revealed six fetuses with a CNV in the 17p11.2 region (Table 2). Case E3640 showed 17p11.2 microdeletion, involving a fragment approxiamtely 3.7 Mb in size and containing 38 OMIM genes, such as RAI1 (Figure 1). Five fetuses (G8354, P587, P3350, P9874, and R425) showed 17p11.2 microduplication. Fetuses G8354, P587, P3350, and P9874 had fragments of about 2.1 Mb containing 21 OMIM genes, such as *RAI1* (Figure 1). Fetus R425 had a fragment approximately 3.7 Mb in size that contained 38 OMIM genes, including *RAI1* (Figure 1).

**TABLE 2:** Results of SNP-array in six fetuses.

Case	SNP-arrary	Size (Mb)	Disease	Inheritance
E3640	arr[hg19] 17p11.2(16,727,490–20,433,723)x1	3.7	SMS	*de novo*
G8354	arr[hg19] 17p11.2(16,567,623–18,743,354)x3	2.1	PTLS	Maternal
P587	arr[hg19] 17p11.2(16,600,022–20,407,931)x3	3.7	PTLS	Maternal
P9874	arr[hg19] 17p11.2(16,600,022–18,746,988)x3	2.1	PTLS	—
P3350	arr[hg19] 17p11.2(16,615,982–18,922,171)x3	2.1	PTLS	*de novo*
R425	arr[hg19] 17p11.2(16,600,022–20,407,931)x3	3.7	PTLS	—

SMS, Smith-magenis syndrome; PTLS, Potocki-lupski syndrome.

### 3.4 Detection of Parent-of-Origin

**FIGURE 2:**
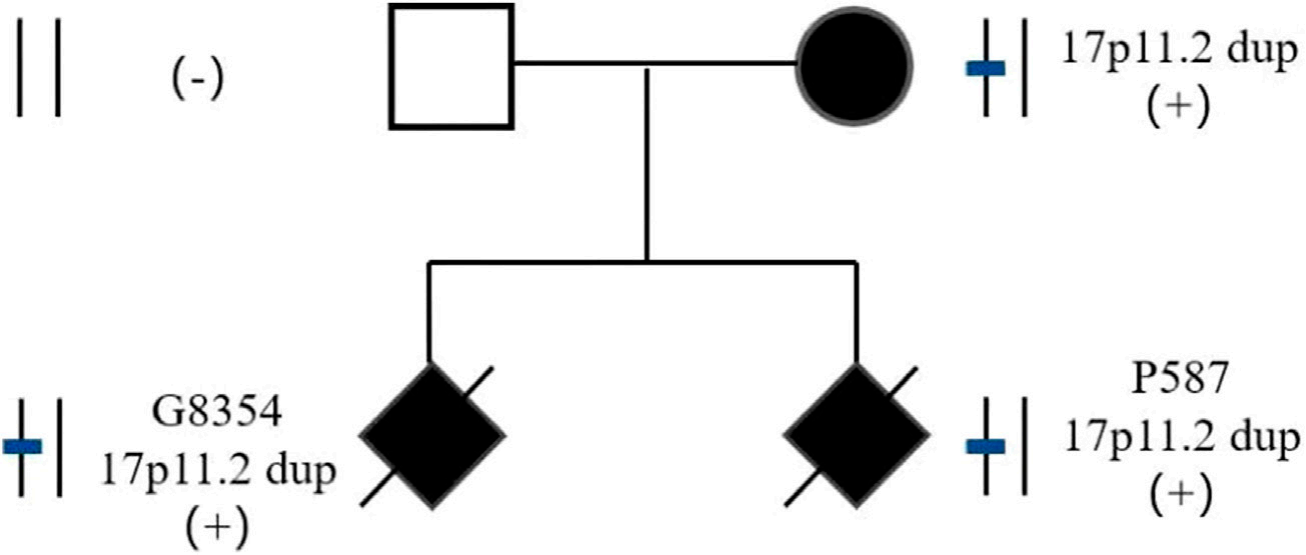
Pedigree of two fetuses in consecutive pregnancies with 17p11.2 duplication.

The parents of two fetuses (P9874 and R425) refused detection of parent-of-origin effects, while the parents of the remaining four fetuses agreed. In case E3640, the microdeletion of CNVs in 17p11.2 was *de novo*. The microduplication of CNVs in 17p11.2 in case P3350 was *de novo*. However, the fetuses G8354 and P587 inherited the CNVs from their mother (Figure 2). G8354 and P587 are from the same family, and their mothers display intellectual disability. G8354, conceived in 2016, was the first child of the mother with intellectual disability, and P587 was the second child conceived in 2020.

### 3.5 Pathogenic Analysis

In fetus E3640, the SNP-array revealed that the microdeletion of the CNV in 17p11.2 involved a 3.7 Mb fragment and contained 38 OMIM genes. According to the database and literature, this segment contained a 17p11.2 recurrent region, including *RAI1*. According to the ClinGen database, this region had haploinsufficiency (score 3), and the loss of heterozygosity in this region was associated with SMS, thus, the clinical significance was pathogenic.

In fetuses G8354, P587, P9874, P3350, and R425, the SNP-array revealed that the microduplication of the CNV in 17p11.2 involved about 2.1–3.7 Mb fragment size and contained 21–38 OMIM genes. According to the database and literature, this segment contained a 17p11.2 recurrent region (including *RAI1*). According to the ClinGen database, this region had a triple dose sensitive effect, which may lead to PTLS, thus, the clinical significance was pathogenic.

### 3.6 Pregnancy outcome

In all six cases the parents of the fetuses chose to terminate the pregnancy after adequate genetic counseling to inform them of the possible risks.

## 4 Discussion

To date, over 100 cases of SMS have been reported worldwide. The incidence of PTLS is rarer, with only over 50 cases reported, and few clinical cases reported in Asia. Both SMS and PTLS are characterized by developmental delays, language disorders, and intellectual disability. SMS also manifests as self-injurious behavior, irritability, sleep disorders, obesity, behavioral abnormalities, and distinct facial and skeletal deformities (Marta et al., 2012; Poisson A et al., 2015; Gupta et al., 2016; Spruyt et al., 2016). SMS is rarely diagnosed in childhood, and its clinical symptoms become more obvious with age (Lee et al., 2012; Gouard et al., 2020). The manifestations of PTLS are less serious than those of SMS. PTLS is characterized by hypotonia, autism, structural cardiovascular abnormalities, and apnea (Lee et al., 2012). These clinical phenotypes are observed in both children and adults.

Currently, there are few reported cases of fetuses with SMS or PTLS with ultrasound phenotypes. This paper summarized the reported cases of ultrasound phenotypes in fetuses with SMS and PTLS (Tables 3, 4). The ultrasound phenotype of one fetus with SMS in this study was cardiac malformation and polyhydramnios, which was consistent with the fetal ultrasound phenotype reported in the literature (Table 3). Congenital heart defects, including ventricular septal defects, have been described in 30% of SMS individuals. Prenatal ultrasound showed that a fetus with cardiac malformation may have cardiac defects after birth. However, since the families decided to terminate the pregnancies, we were not able to track whether the fetuses were characterized with cardiac defects and other phenotypes.

**TABLE 3:** Reports of ultrasound phenotypes in fetuses with SMS.

References	Ultrasound phenotypes in fetuses
Thomas et al. (2000)	Short heads, short bones and heart malformations
Fan and Farrell (2010)	Duplicated right ureter
Zhou et al. (2015)	Ventricular septal defect, pulmonary stenosis, fetal growth restriction
Lei et al. (2016)	Increased nuchael translucency, mild lateral ventriculomegaly, and congenital heart defects
Zhang et al. (2020a)	Polyhydramnios, ventriculomegaly and external genital defects

**TABLE 4:** Reports of ultrasound phenotypes in fetuses with PTLS.

References	Ultrasound phenotypes in fetuses
Popowski et al. (2012)	Asymmetric ears
Bravo et al. (2013)	Hypoplastic left heart and aberrant right subclavian artery
Dhanaraj et al. (2015)	Bilateral clubfoot
Yusupov et al. (2011)	Micrognathia, increased nuchal translucency, intrauterine growth retardation, and a two-vessel cord

Of the other five fetuses with PTLS, two had cardiac malformations, two had normal ultrasound phenotypes, and one had minor ultrasonographic markers. These are inconsistent with the literature (Table 4). In this study, two fetuses with PTLS were the first and second fetuses of the same mother with intellectual disability. The ultrasound phenotype of the first fetus was cardiac malformation and fetal growth restriction, while the ultrasound phenotype of the second was normal. Based on these reports and our cases, the prenatal ultrasound findings of PTLS are not specific and may be completely normal. The most frequent ultrasound findings in fetuses are cardiovascular anomalies and are reported in about 40% of individuals with PTLS. The ultrasonic phenotypes of these cases may enrich the clinical database. Clinical phenotypes of SMS and PTLS are complex and diverse, requiring a large amount of sample data and more in-depth clinical studies.

With the development of molecular diagnostic technology, an increasing number of microdeletion and microduplication syndromes have been found (Wou et al., 2016); in the present study, SMS and PTLS were two such syndromes. Chromosome karyotype analysis is the most classical and common detection method, but the resolution is low, and it can generally only detect duplication, deletion, translocation, and other structural abnormalities larger than 10 Mb. The karyotype analysis of six fetuses in this study was normal. Currently, cases of SMS and PTLS are detected using chromosomal microarray analysis (CMA). CMA is divided into two categories (Brady and Vermeesch, 2012), array-based comparative genomic hybridization (array-CGH) and single-nucleotide polymorphism arrays (SNP-array) (Xiang et al., 2020). SNP-array can not only detect the CNV, but it can also detect abnormalities such as uniparental disomy, low level chimera, and triploidy (Zhang Y. et al., 2020). In this study, the SNP-array showed that one case of SMS and one case of PTLS had a 3.7 Mb microdeletion and a 3.7 Mb microduplication, respectively, in the same location. In general, cases with microduplications have milder clinical manifestations than cases with microdeletions. In this study, one case of SMS with a 3.7 Mb microdeletion had a severe ultrasonic phenotype, while one case of PTLS with a 3.7 Mb microduplication had a normal ultrasonic phenotype. A 2.1 Mb microduplication was observed in four cases of PTLS. The region of genetic variation in these all cases contained the *RAI1* gene. RAI 1 is a transcription factor involved in cell growth and cell cycle regulation, bone and bone development, lipid and glucose metabolism, embryonic neural development and neuronal differentiation, behavioral function, and circadian activity (Vilhais-Neto et al., 2010; Fragoso et al., 2015; Garay et al., 2020). Copy number loss of *RAI1* results in SMS (Falco et al., 2017), and copy number gain of *RAI1* results in PTLS (Chen et al., 2016).

In this study, three of the five fetuses with PTLS had mothers with intellectual disability. One fetus’ parents refused verification, and the other two fetuses were the first and second fetuses of the same mother with intellectual disability, and the results of detecting the parent-of-origin showed maternal inheritance. To improve the diagnosis rate of abnormal fetuses, karyotype analysis and SNP-array should be recommended for pregnant women with intellectual disability. The SNP-array of one fetus with SMS and one fetus with PTLS identified the origins as *de novo* by detecting the parent-of-origin. SMS and PTLS can be inherited in an autosomal dominant manner, however most are *de novo*, and very few patients inherit the aforementioned syndromes from their parents. For *de novo* CNV-induced SMS and PTLS, the risk of recurrence was less than 1%, but prenatal diagnosis was still required for a second pregnancy. Currently, there are few reports of births in patients with SMS and PTLS (Acquaviva et al.; Magoulas et al., 2014). In the case of the mother with PTLS reported in this paper, the risk of offspring recurrence was 50%. Considering that both the first and second children had PTLS, it is suggested that the patient should have prenatal diagnosis or undergo third generation *in vitro* fertilization to ensure healthy children.

The prenatal ultrasound phenotype of 17p11.2 copy number abnormalities associated with Smith–Magenis syndrome and Potocki–Lupski syndrome in fetuses displays heterogeneity. There are many challenges in fetal ultrasound diagnosis, which can be specifically divided as follows: 1) many structural abnormalities may be discovered in the middle and late stages; 2) some phenotypes, such as intellectual disability, cannot be expressed during the fetal period; 3) reduced fetal movement and other functional phenotypes have great limitations; 4) the prenatal phenotypes of chromosomal and other genetic disorders were heterogeneous and nonspecific in clinical descriptions, even in fetuses. We believe that fetal magnetic resonance imaging plays a good auxiliary role in the detection of nervous system abnormalities (Di Mascio et al., 2021; Papaioannou et al., 2021).

In conclusion, the 17p11.2 copy number abnormality has variable expressivity with the phenotypes being milder in some patients. SNP-array analysis is used in the prenatal diagnosis of fetal ultrasound abnormalities and pregnant women with intellectual disability. In the future, detailed molecular genetic testing and prenatal ultrasonographic analysis of a higher number of cases of SMS and PTLS in fetuses will aid in the identification of SMS and PTLS in fetuses and in the accurate localization of other related genes.

## Data Availability

The raw data supporting the conclusion of this article will be made available by the authors, without undue reservation.
